# Perceived Benefits of Curling in Older Canadian Women

**DOI:** 10.1093/geronb/gbae041

**Published:** 2024-03-23

**Authors:** Alia Mazhar, Shruti Patelia, Joseph Baker

**Affiliations:** Faculty of Kinesiology and Physical Education, University of Toronto, Toronto, Ontario, Canada; Faculty of Kinesiology and Physical Education, University of Toronto, Toronto, Ontario, Canada; Faculty of Kinesiology and Physical Education, University of Toronto, Toronto, Ontario, Canada

**Keywords:** Competition, Social connection and support, Sport

## Abstract

**Objectives:**

An increasing proportion of older Canadians are pursuing sports. The objective of this study was to examine the experiences of older Canadian women in curling.

**Methods:**

Semistructured interviews were conducted with 17 participants. Interviews were transcribed and coded using thematic analysis.

**Results:**

Participants simultaneously resisted, accepted, and ultimately redefined conceptualizations of personal aging in the context of curling. Although gender was deemed inconsequential, concepts such as the physical and cognitive demands of curling, inclusivity, social connection and support, and competition were perceived to be integral to participants’ experiences.

**Discussion:**

This study extends our understanding of the value of curling specifically, and sport in general, for older women.

The proportion of older Canadians (generally defined as 65 and above) is rising, and older adults are projected to make up more than 25% of the overall population by 2068 (Statistics Canada, 2022). In addition to the complexities of population aging, factors such as gender can shape an individual’s experience. Gendered aging positions women as facing greater negative consequences in old age in comparison to men; for example, women diagnosed with chronic disease experience illness for longer periods of time (due to a longer life expectancy) compared to men (Backes et al., 2006). From a sociocultural perspective, women also differ from men in their gendered roles (e.g., caregiving, domestic duties), careers (e.g., disruptions in their career due to childbearing and rearing), and their ability to retire, which influences their financial capabilities during old age (Cepellos, 2021; Krekula, 2007). Women face added pressures such as to age well (aesthetically and physically; Pilcher & Martin, 2020) and continue in their caretaking roles (i.e., take care of ailing spouses, grandchildren, and their own parents; Backes et al., 2006; Pike, 2015). Although it is clear that women experience aging differently than men, it is surprising how little we know about their aging experiences.

A promising area to understand how women negotiate their aging process is sport. The increase in the aging population has coincided with an increase in the number of older adults participating in sports, an activity offering a range of physical and psychosocial benefits. Although sports participation occurs at many levels, researchers have devoted considerable effort to studying competitive Masters Athletes, individuals who are typically 35 years of age and compete in organized sports (Makepeace et al., 2021). According to a recent scoping review, most work examining Masters Athletes has been conducted in the areas of physical or physiological health and/or maintenance of performance, whereas psychological and social elements remain understudied (Patelia et al., 2023). Moreover, we lack a clear understanding of women’s experiences, particularly older women, in sports (Pike, 2015). For instance, there are three times more studies focusing exclusively on male as opposed to female athletes (Patelia et al., 2023). Furthermore, studies using older mixed samples make it difficult to identify women’s unique experiences. This has resulted in a small, albeit expanding, literature base on the value of sport for older women as a population.

Current literature suggests that organized sports can provide unique opportunities for experiencing positive psychosocial outcomes for older women (West et al., 2019). For instance, studies of older women participating in volleyball reported increased feelings of belongingness, experiences of enhanced social support, enjoyment through competition, and value in trying new activities (Kirby & Kluge, 2013, 2021). Similarly, engagement in softball is associated with greater overall well-being, social embeddedness, pride in athletic ability, and providing an opportunity to counter gender stereotypes (Liechty et al., 2016; Wong et al., 2019). Research in field hockey suggests this sport can facilitate social support, friendship, and community (Litchfield & Dionigi, 2013). In one study of older women in the sport of lawn bowls, researchers discovered that this cohort prioritized competition and the social aspect of the sport (Heuser, 2005). Finally, older women taking up swimming report reduced anxiety and stress, and higher self-esteem and quality of life (Oliveira et al., 2019). Multi-sport studies re-affirm these observations and suggest sports can be used by older women to develop athletic identities and partake in identity management, challenge stereotypes of the aging female body, find/create meaning, and negotiate the aging process (Litchfield & Dionigi, 2012; Pfister, 2012). Expanding on the latter, sports can be used by older adults to resist the negative effects of aging (e.g., physical decline, physical inactivity, and social isolation), while accepting and adapting to age-related limitations, thereby redefining the aging process (Dionigi, 2010; Dionigi et al., 2013; Horton et al., 2018). Collectively, these psychosocial factors are reported to influence one’s motivation to maintain sports engagement (see Brown, 2019; León-Guereño et al., 2021).

Although research on older women in sports is emerging, we currently have insufficient evidence to draw conclusions on sports experiences for *all* women and *across* all sports. However, we have a unique opportunity to study older women’s experiences in sports since women’s involvement in sports is rising. For example, in the 2017 World Masters Games 47% of all athletes were women (International Masters Games, 2017). Increased participation rates in conjunction with the likelihood that older women will experience the negative effects of gendered aging, the psychosocial benefits associated with sport-participations become all that much more relevant and desirable. As such, studying older women’s experiences in sports should be a priority.

One sport that has been adopted by older women is curling, a popular winter team sport that is played around the world and has a large uptake in countries like Canada (Mair, 2009). For example, Curling Canada (2019) estimated that in 2015 approximately 1.5 million Canadians curled, 20% of whom were between the ages of 50 and 64. As a winter sport, it offers the opportunity to participate in sport-related competitions (and physical activity; PA) in the winter months for those living in the northern hemisphere. Playing requires minimal equipment and relies on players for self-regulation (Tate, 2011). Values of sportsmanship, socialization, building community, patience, skill, and volunteerism are prized within the curling community (Allain, 2020; Brooks et al., 2017; Mair, 2009; Tate, 2011). Surprisingly, this sport has received little attention from researchers exploring the role of sport in older populations.

Historically, the sport has been inclusive of fitness and skill level and women’s participation steadily become normalized (Tate, 2011). From this perspective, curling’s greatest contribution is its acceptance of aging bodies (although this may be in flux due to the professionalization of the sport, see Allain, 2020 and Allain & Marshall, 2020). Curling facilitates the acceptance of older bodies by creating a tangible space for socialization and building community and through the modifications it has introduced in the sport. Examples of adaptations include stabilizers, stick curling, wheelchair curling, and vision-impaired curling. Ultimately, these additions allow novices to enter the sport and enable individuals with health and functional limitations to maintain their engagement. Thus, curling is unique in that it is highly inclusive of age and facilitates participation from an aging body. Given that aging, invariably, affects physical function and curling provides an avenue to compensate for these changes, curling becomes a distinct site to explore the intersection of aging and sport. As such, the study of curling from the perspective of older adults and older women may lead to valuable insight.

A research base is developing in areas of curling and coaching (Cormack & Gillman, 2021; Paquette & Sullivan, 2012), its relationship to nationhood (Reid, 2010), and its psychophysical benefits (Kim et al., 2017; Stone et al., 2018). From a psychosocial perspective, Allain (2020) and Allain & Marshall (2020) have studied the experiences of older men who curl. They outline how this population negotiates ideas of aging and masculinity, acceptance of functional limitations in sports, values community and friendship, and the importance of maintaining physical activity. These studies provide knowledge on the role of sport among older adults (particularly among men); however, the experiences of women are largely missing. Only two studies focus on women’s participation in the sport recreationally (Leipert et al., 2011, 2014). In both studies, building meaningful connections was integral to the curling experience, and curling positively affected women’s biopsychosocial health which contributed to their overall wellbeing. However, participants’ ages ranged from 12 to 75 years and therefore conclusions about the experiences of older Canadian women cannot be drawn. Although it stands to reason that curling may provide a myriad of benefits for older adults, research in this area, particularly among older Canadian women, is limited (Stone et al., 2018).

It is important to learn about their experiences because (a) we know relatively little about this phenomenon, (b) this knowledge may help inform future policies and resource allocation, and (c) shapes the way sport is promoted to this demographic. Furthermore, examining the participation of older women in a wide array of sports is crucial to understanding how different sports contribute to the lives of older women. The present study focuses on curling as both anecdotal and literary evidence highlight the popularity of the sport among older adults in Canada; for example, Curling Canada, the governing body of curling, estimated that in 2015 approximately 1.5 million Canadians curled, 20% of who were between the ages of 50 and 64 (Potwarka & Wilson, 2015). For these reasons, this project explored participation experiences of older, Canadian women in the sport of curling, with a particular focus on participants’ perceived benefits of their participation.

## Method

This study included participants aged 50 and above. This age cut-off was chosen (a) due to the lack of consensus regarding the age at which adults are considered “older adults” (Jenkin, 2020), (b) limited understanding of athletes during periods of midlife, and (c) the intrinsic value of understanding these participants’ perceptions given the lack of information on groups in mid-life. This study received institutional ethics approval and written informed consent was obtained from all participants. Participants took part in a 1-h, semi-structured interview conducted virtually through the video conferencing software, Zoom (Version 5.5.0), due to the coronavirus disease-2019 (COVID-19) pandemic.

Initial calls for recruitment were posted to social media sites (e.g., Twitter and Facebook). In addition, a contact was made at a curling association in Ontario, who then shared study information with their personal and professional acquaintances. There was also an element of snowball sampling as some interviewees recommended their friends for the study. Recruitment occurred from January to March in 2021 and all individuals who met the study criteria were invited to participate. In total, 17 interviews were conducted with Canadian women between the ages of 53–75 (average age of 62.47 [±6.53]). Four women identified as East Asian, and the remainder identified as Caucasian or European (Table 1). Most of the participants were married and eight women had children. In addition, most had some level of college or university education, making this group highly educated. More than half the women in this sample (*n* = 12) were retired. Half of the sample self-identified as competitive curlers.

**Table 1.** Demographic Information of Participants

Name	Age	Marital status	Children	Province of residence	Highest level of education^a^	Employment status	Player identification
Amanda	60	Married	0	Ontario	2	Retired	Competitive
Beth	56	Married	0	Ontario	2	Employed, full-time	Recreational
Betty	58	Single	0	Ontario	1	Retired	Recreational
Camille	62	Married	1–2	Ontario	7	Retired	Recreational
Cindy	60	Common Law	0	Manitoba	4	Retired	Competitive
Claire	53	Separated	0	Manitoba	4	Employed, full-time	Competitive
Elizabeth	73	Married	1–2	Ontario	2	Retired	Recreational
Gabriella	64	Married	1–2	Ontario	1	Self-employed, semi-retired	Competitive
Giselle	67	Married	0	Ontario	1	Retired	Competitive
Jade	64	Separated	0	Ontario	3	Retired	Competitive
Jennie	70	Married	1–2	Ontario	3	Retired	Competitive
Joanne	60	Married	3–4	Ontario	3	Retired	Recreational
Kate	63	Married	0	Ontario	6	Retired	Recreational
Lilly	56	Married	1–2	Ontario	2	Employed, full-time	Competitive
Penny	75	Widowed	1–2	Ontario	5	Retired	Competitive
Summer	53	Common Law	1–2	Manitoba	4	Employed, part-time	Competitive
Teri	68	Married	0	Ontario	3	Retired	Recreational

*Note*:

^a^1 refers to Master’s degree, 2 refers to Bachelor’s degree, 3 refers to College degree, 4 refers to trade/technical/vocational training, 5 refers to a university diploma, 6 refers to some university, and 7 refers to high school.

### Theoretical Foundation

Critical realism (CR) is a theoretical perspective founded by Roy Bhaskar (Fletcher, 2017; Gorski, 2013). Critical realists agree that phenomena exist in the “real world” (i.e., objects and structures are not merely “constructions” or “interpretations” of individuals; Ryba et al., 2020). Importantly, CR rejects the notion of “multiple realities” based on individual experiences, as argued by proponents of constructivism, and instead argues that operation in the same reality can lead to diverse and valid perspectives (Wiltshire, 2018).

Critical realism (CR) argues for a separation between ontology and epistemology (Fletcher 2017; Wiltshire, 2018). Ontology is separated into three interacting layers: empirical (events that are experienced), actual (events that occur irrespective of them being experienced by individuals), and the real (casual mechanisms behind the occurrence of events; Fletcher, 2017; Ryba et al., 2020). Given the separation of ontology and epistemology, critical realists can draw from a range of methodologies (e.g., regression analysis, interviewing, ethnography, etc.; Wiltshire, 2018).

An important distinction between CR and other theoretical perspectives is that CR attempts to explain, rather than simply describe, the phenomena under investigation (Fletcher, 2017; Ryba et al., 2020; Wiltshire, 2018). To this end, many critical realists accept the Transformational Model of Social Activity (TMSA) as a means to represent and explain social reality. The TMSA was originally proposed by Bhaskar (1978) (expanded by others such as Faulkner and Runde, 2013) and focuses on human agency, social structure, and the relationship between these concepts. In essence, human agency is guided and constrained by social structures and human actions unconsciously reproduce these structures.

With humility, CR accepts that the knowledge generated by scientists is fallible as it is produced through imperfect methods (e.g., sensory, discursive, experimental; Ryba et al., 2020; Wiltshire, 2018). From this perspective, this study takes place in the “real” and it is accepted that our understanding of the phenomena may be incomplete. Moreover, as CR is concerned with learning about the way in which phenomena behave in the social world, attention was paid to understanding how aspects of the curling experience affect the lives of these women. For example, if participants derived social benefits from sports participation, did that affect their experience of aging, if so, how?

### Analysis

All interviews were audio recorded using Zoom and a backup recording was created using a phone recorder. Interviews were transcribed verbatim using an online transcription program. The primary author then went through each transcript to ensure accuracy and increase familiarity with the data. All transcripts were sent to participants for their edits and approval. Transcripts were then anonymized using pseudonyms. Interviews were conducted by the first author and debriefs were conducted with the senior author following the interviews. Data was analyzed using thematic analysis as proposed by Clarke and Braun (2013). Authors independently familiarized themselves with the data which involved multiple read-throughs of transcripts with audio recordings. This was followed by an inductive approach to coding, which meant that the identified themes were closely tied to the data. The first author manually coded each transcript by tagging data items of interest. This process was repeated twice to produce 349 unique codes which were then grouped into three higher order themes. During this process, the technique of a “critical friend” was utilized allowing researchers to collaborate, challenge one another, and ultimately come to a consensus.

## Results

The purpose of this study was to explore the experiences of older Canadian women participating in curling. The participants from this study used sport (and PA) to facilitate a nuanced perspective of aging whereby they simultaneously resisted, adapted to, and redefined the aging process.

### Resisting Aging: A Desire to Age Well

Participants used curling as a context to resist the negative aspects of aging from a physical and mental standpoint as discussed in the subthemes of controllable factors and chess on ice.

#### Controllable factors

Participants believed that PA overall, and curling specifically, allowed them to control certain aspects of aging so that they could delay this process. For example, it was believed that the negative effects associated with aging can be delayed through PA. Lilly explained, “I think as you get older your body parts tend to rebel against you. I’m hoping that doesn’t really happen, doing all that fitness and working out, hoping that it slows … down anything that could happen.” Similarly, Betty expressed her desire to resist elements she linked to the normal aging process:

I’m not afraid of dying. I’m afraid of not aging well … I’m really afraid of Parkinson’s disease. I want to stay as healthy and as fit as I possibly can so that I can ward off any of these degenerative diseases as much as possible.

Although this group of women were participants in this study due to their engagement in curling, very few of them referred specifically to curling to delay or resist the process of aging. Instead, the terminology used by participants (i.e., fitness, exercise, and PA) indicated a broader goal of using all aspects of PA to exert control to delay/resist the aging process.

Participants frequently used the saying “use it or lose it,” as Amanda explained, “I mean the adage, if you don’t use it, you lose it, is very true and that’s a principle that you have to always be mindful of.” Thus, implying that work was required to keep certain factors related to the aging process under control and if “it” was lost, women would be at risk of experiencing the negative aspects associated with aging. When it came to using “it,” the women in this study did not have an all-or-nothing approach, rather they maintained that it was important to adjust to limitations and remain active, as Amanda explains:

[Y]ou have to be a little mindful of that [limitations] and be a little more careful, but at the same time, not sit back and do nothing. I do have friends who have done less and less and less and then they find themselves not being able to do much.

#### Chess on ice

Jade described curling as, “chess on ice” indicating that it requires a level of cognitive engagement. This point was elaborated upon by individuals like Lilly who explained, “there’s a lot more analysis in curling” and Claire who said, “it’s very challenging … both mentally and physically.” Like chess, curlers strategize to anticipate their opponents’ throws and hypothesize their responses. Moreover, the strategy must be dynamic to adjust for the (un)successful deliveries of rocks by both teams. In addition, women reported that curling allowed them to enhance their focus and emotional regulation skills. As Penny said, “mentally it keeps you sharp.” Curling was used to (sub)consciously maintain cognitive abilities in the face of aging.

### Acceptance and Adaptation: Managing the Inevitable Parts of Aging

Paradoxically, and despite the experiences of some participants who actively attempted to control their aging experience (as discussed earlier), women accepted that getting older was just another phase in life. For instance, they described this period as one of new challenges and changes including the passing of loved ones, grappling with mortality, and (some) physical and cognitive decline. Importantly, all participants had a positive attitude toward sports participation at this stage of their lives and used this to adapt to the aging process.

#### Curling for all

This sport was described by participants as particularly “welcoming” as Joanne noted, “I don’t know whether other sports have such an active community of and welcomes their seniors like curling does.” In addition to the friendly nature of the sport, curling was perceived by most individuals to be inclusive of fitness, body shape/physique, and athletic ability. Importantly, curling was described as an accessible sport as Giselle explained, “there are all kinds of adaptations in the sport of curling, that allow you to curl till you’re 100 or more.” Given the accessible nature of the sport, curling was positioned as a tool to accept and adapt to such limitations, as evident through Amanda’s narration:

[T]he introduction of stick curling … allowed people to stay curling, and to stay in the game and to stay with their friends. My father, he lost his vision and was legally blind, and he went to stick curling and … he played till he was almost 90 years old, because he could still go, and it keeps people in those communities, and it keeps them involved, and it’s important.

Moreover, participants were willing to accept the modifications offered by the sport and some had already switched to using the stick. Additionally, these women found that they could still feel successful, find joy in the sport, and remain socially engaged. The adaptations present in the sport of curling allow participants to readily adjust for factors related to the aging process outside of their control.

#### Social connection and support

Curling was described as a venue to expand social networks. For instance, Summer described, “curling has grown my social circle, like I would say that half of the people that I associate with are curling people.” Camille and others highlighted the value of curling and curling clubs for helping them meet new people, particularly during retirement when opportunities for social interaction are more limited. Others emphasized how curling brought them “lifetime friends,” with Kate recalling creating friendships that might never have been, had it not been for curling.

Several participants spoke of the social support they both received and provided in these friendships; for example, Joanne recalled recently participating in cooking meals for one of her curling friends whose husband had fallen terminally ill. Others, like Amanda, narrated incidents of support they had received, “I’ve had and have very good friendships through curling. So, when I’ve had difficult points in my life, when there’s been milestones, positive milestones, sad milestones, it’s those friendships that have been there for me.” Moreover, during the COVID-19 pandemic, these relationships provided an avenue of support. For example, if they could not meet at the rink, they would connect over video chat to discuss curling, play games, and remind one another to remain hopeful for the next curling year. As noted by the participants, social connection and social support were integral aspects of the curling experience for these women.

### Redefining Aging: New Conceptions of Aging

Participants’ conceptualization of age as “just a number” (Claire) emphasized elements of aging as a complex and nuanced mindset. In our sample, women redefined older adulthood as a period of learning, new experiences, building new connections, and “living life to the fullest.” For example, Joanne explained, “I’m not afraid to do new things because I am over 60. I’m not afraid to put myself out there.” The following subthemes further our understanding of how redefinition of aging occurred for this cohort.

#### The value of competition

Early in her interview, Summer explained, “Okay, so [competition is] probably first and foremost and secondary is the friendships and the camaraderie. *Funny, it’s not first. First is actually going out and playing the game.* [emphasis added].” This was an apt description of the importance of competition for many participants in this study who pursued curling as an opportunity to compete against others in their age category. For example, Betty explained, “To me curling is the one sport where it’s better to be older because you can participate against older people, and maybe win.” In addition to competing, achieving sporting success was an important element of the experiences of these curlers.

Participation in curling at this stage of their lives expanded the activities that older women can and should participate in. In fact, some women aspired to reach specific age milestones as this would qualify them to compete in competitions like the Masters or Grandmasters. Jennie explained, “My age has never held me back. At a time when my peer group is stepping back from competitive curling, I am still improving and playing better and better than many curlers in my league.” For this group, aging, gender, and competition were not viewed as contradicting ideas.

#### Aging as women

This study included several questions for participants to reflect on the possible impact of their gender on their participation in curling. Most women (*n* = 13) believed their gender had no bearing on their curling participation or experience other than dictating the types of leagues they were eligible to participate in. Betty explained,

No, the only way it [gender] impacts me is it defines what leagues I can and cannot participate in. We’ve got a ladies’ league, a skins open league, and then the mixed league, you have to have two women and two men. Although I’d like to play with, these people, I can’t, because they can’t have the extra woman – that type of thing. That’s still the only way it’s impacted me, just in terms of what leagues I can participate in. But otherwise, I would never have even considered gender.

Despite this being the consensus of the group, some women described gender norms that sometimes affected their participation. For example, men were more likely to get preferential ice times and were more likely to be chosen as the skip (captain). However, participants explained that these aspects were in a state of flux, meaning more clubs now gave equal access to men and women and teams were more likely to pick a skip based on merit.

## Discussion

The key themes from this study reflect a complex perspective of aging and lend support to other literature (see Dionigi et al., 2013). Although studies focusing on older women in sport have outlined how participants resist aging (see Dionigi, 2010; Litchfield & Dionigi, 2013), our work highlights the nuanced experiences of women in curling, compared to the findings of Dionigi and colleagues (2013) who concluded that individuals primarily referenced sport to resist the aging process. Older women in this study saw the cessation of physical and mental stimulation as placing them at risk of becoming sedentary, experiencing physical and mental decline, reducing their performance in curling, and leading to social isolation. Dionigi (2006) found similar results as participants equated the termination of PA to a decreased quality of life as they would lose their independence, health, and sense of control. Similar to propositions by Baker et al. (2010), the responses from participants in this study suggested their current experience of aging would drastically change if they were inactive.

Current aging discourse positions sport as a tool to address age-related decline (Gard et al., 2016). The management of the aging process, to an extent, has been transferred to the individual whereby it is their responsibility to engage in PA to age well and reduce burdens on others and the health care system (Pike, 2011). In other work, Masters Athletes have internalized this trope, considering aging well a choice free of structural constraints and viewing sedentary individuals as irrational (Gard et al., 2016). Compared to older men, older women are expected to retain a youthful and traditionally feminine appearance (Chonody & Teater, 2015). Operating in this societal context, it is understandable why women in this study outright resist aging and use their autonomy and resources to engage in behaviors (i.e., curling and PA) that will help them age well, both functionally and aesthetically.

Previous literature suggests that to start or maintain sports engagement, older women adapt to their functional limitations by modifying their sport participation or sport type (Dionigi, 2010; Horton et al., 2018; Liechty et al., 2016). Furthermore, modifications that lead to adaptation can facilitate the acceptance of aging (Dionigi et al., 2013) and allow individuals to reap the benefits associated with sport. Curling is unique in the number of adaptations it offers; therefore, it is possible that in comparison to other sports, curling can more easily facilitate the adaptation to and acceptance of older age. In this study, it seemed that women readily accepted the use of curling modifications whereas elsewhere older men seem to be more reluctant (Allain & Marshall, 2020). It may be relevant to consider that the uptake of modified curling may be stronger among older women; however, more research is needed to confirm this assertion. Considering the aging population in many countries, it is important to deepen our understanding of sports which can facilitate the adaptation to and acceptance of aging so that individuals can continue to gain the benefits associated with sport.

Older adulthood is often depicted as a time of loneliness and disengagement (Litchfield & Dionigi, 2013), with heightened risks for older women (Davidson et al., 2011). However, for these women participation in curling has transformed older adulthood into a period to pursue meaningful social connections. This is similar to work showing older men enjoy the capacity of curling to facilitate social connection (Allain, 2020; Allain & Marshall, 2020). Social support can allow older adults to manage challenging circumstances, such as disease and loss, more effectively (Yu & Liu, 2021). Thus, participation in curling can provide a protective factor against loneliness and act as a tool for women to better navigate older adulthood. Although there is a common perception of curling as a social sport (see Allain, 2020; Mair, 2009), this study extends the existing literature by emphasizing the unique opportunities sport provides for continued development for this demographic.

In the context of gendered aging, it is remarkable that women in this cohort sought to change aging narratives (i.e., redefine aging) through their participation in curling (and to an extent PA). For this cohort, aging represented a time of renewed vitality marked by a need to maintain cognitive and functional abilities. Some factors appear to contribute toward the act of redefining aging. First, having worked earlier in life allows women to be better physically, financially, and socially adjusted in later life (Backes et al., 2006; Krekula, 2007). As most women in this sample were highly educated, had careers, and were living with their spouse/partner (i.e., dual pension/income), it is likely that they had the material resources to pursue PA/curling. Furthermore, women (apart from one individual) had either rekindled or continued their curling participation. By these accounts, the women in this study carried privileges (i.e., financial resources to pursue curling and earlier exposure to sport which may facilitate the adoption of sport in later life), and it is unknown whether older women without these privileges would be able to redefine their own aging narratives in the same capacity.

As the women in this study redefined aging, in some ways their experiences mirrored those of men. For instance, older adulthood affords men the freedom to pursue interests and connect with social contacts outside the home (Backes et al., 2006). Similarly, women in this study had the freedom to pursue curling and engage with their curling network. Furthermore, older adults have been encouraged to pursue leisure PA rather than competitive sports (Gard et al., 2016). However, as seen elsewhere (Dionigi, 2010; Litchfield & Dionigi, 2012; Pfister, 2012) women in this study have changed their aging narratives to include competitive sport participation. Some (Baker et al., 2010) have suggested that when PA is promoted to older adults, the critical element of competition is missing, which may affect the number of older adults who then choose to become active. Participation in curling can act as a tool to redefine personal narratives of aging and act as a protective factor against the gendered aspects of this process.

Over time, legislation has sought to promote equality in women’s sports (e.g., the 1968 Canadian Policy on Women in Sport). When women in this study were growing up, Canadian society, and in turn sporting structures, was undergoing a shift to remove barriers for women in sport, the effects of which are evident in this study (e.g., equal access to preferred ice times, choosing leaders based on merit as opposed to gender). However, it was notable that women in this study did not characterize gender as a barrier to their sport participation. We theorize that part of this may be due to structural changes in the sport and the ability of women to exercise their agency with respect to sports participation due to their social standing (e.g., belonging to the dominant ethnic and social class that participates in curling). In addition, women explained that during child-rearing years they would bring children to the rink when childcare activities were available. During work-oriented years, they participated in leagues in line with their working schedules. In sum, the women in this study were able to exercise their agency throughout different periods of life to curl. It is unclear whether these women engaged in advocacy to change structures in curling; however, it is apparent that by exercising their agency to pursue curling at this stage of their lives, they are (un)consciously reproducing norms about who can engage in sport and when.

## Limitations

Although this study is informative, there are some limitations that need to be considered. Only English-speaking participants were recruited and given that Canada is a bilingual country, perspectives from French-speaking may have been missed. The data collection for this study took place during the COVID-19 pandemic, at a time when curling rinks were closed or offered limited access (i.e., access to the ice once or twice a week). Thus, the timing of data collection could have affected participants’ responses in an overly positive and/or negative way.

In line with previous literature (see Wright, 2012), our sample was predominantly White and highly educated. Interestingly, our work suggests that Masters Athletes may have unique characteristics surrounding childbearing. For example, less than half of the participants were mothers which is less than the proportion of women in Canada who are mothers (Statistics Canada, 2012, 2017). This may have implications for the type of older woman who is more likely to engage in curling.

Furthermore, the present study included individuals who self-identified as recreational or competitive players. The term “competitive” was not defined for the interviewees and given the range of interpretation, we chose not to dichotomize the sample based on this characteristic. However, the element of competition was found to play an important role for most participants, therefore, future work should consider exploring how sport experiences of recreational and competitive athletes may differ. In terms of life stages, older adulthood is not clearly defined and including individuals who are closer to 50 may have affected the results. In the opposite direction, women over the age of 76 did not participate in this study; therefore, the experiences of women participating in curling at older ages were unintentionally excluded.

## Conclusion

Our findings extend a budding literature base on the effects of sports participation on older women. The results provided several intriguing findings regarding how older women use sport, and perhaps PA more broadly, to simultaneously resist, accept, and redefine their aging process. Our work suggests curling can provide “protection” against components of gendered aging and future work should address this aspect in curling and other sports. Given the unique nature of sports participation versus exercise more generally, future research should further explore the role of competition in older women’s participation in sports. If these results point to a general value of involvement in competitive activities, in particular, there may be important implications for PA recommendations for older women. Finally, the capability of sport (in this case curling) to positively affect the aging process is intriguing and may have implications for the study of sport and aging more generally.
